# Machine Learning Prediction of Anterior Open‐Bite Development After Stabilization Splint Treatment in Temporomandibular Disorder

**DOI:** 10.1002/cre2.70447

**Published:** 2026-08-30

**Authors:** Jae Joon Hwang, Hye‐Mi Jeon, Hye‐Min Ju, Sung‐Hee Jeong, Yong‐Woo Ahn, Soo‐Min Ok

**Affiliations:** ^1^ Department of Oral and Maxillofacial Radiology, Dental and Life Science Institute, School of Dentistry Pusan National University Yangsan Republic of Korea; ^2^ Dental Research Institute Pusan National University Dental Hospital Yangsan Republic of Korea; ^3^ Department of Oral Medicine Pusan National University Hospital Busan Republic of Korea; ^4^ Biomedical Research Institute Pusan National University Hospital Busan Republic of Korea; ^5^ Department of Oral Medicine, Dental and Life Science Institute, School of Dentistry Pusan National University Yangsan Republic of Korea

**Keywords:** machine learning, occlusal splint, open‐bite, temporomandibular disorders

## Abstract

**Objectives:**

To develop interpretable machine learning (ML) models for risk stratification of clinically meaningful anterior overbite decrease after stabilization splint therapy in temporomandibular disorder (TMD) patients, and to compare ML performance with conventional logistic regression.

**Materials and Methods:**

This retrospective cohort included 87 TMD patients treated with stabilization splints, physical therapy, and pharmacologic therapy. Thirty‐five pre‐treatment cephalometric and demographic features were used. The primary outcome was overbite difference (OD = post‐treatment minus pre‐treatment overbite). For classification, patients were labeled as Decrease (OD < −0.5 mm; *n* = 37) versus Non‐Decrease (OD ≥ −0.5 mm; *n* = 50), combining No Change and Increase due to severe imbalance in the Increase subgroup. Six classifiers (logistic regression, random forest, extreme gradient boosting (XGBoost), Light Gradient Boosting Machine, support vector machine, k‐nearest neighbors) were evaluated using stratified fivefold cross‐validation and leave‐one‐out cross‐validation. Class imbalance was handled using class‐weighting and the Synthetic Minority Over‐sampling Technique (SMOTE) applied within the cross‐validation loop. For regression, nine algorithms were tested to predict continuous OD. Model explainability was assessed using SHapley Additive exPlanations (SHAP).

**Results:**

In fivefold cross‐validation, XGBoost achieved the best discrimination (AUC = 0.734), outperforming logistic regression (AUC = 0.611). With SMOTE, the random forest achieved a comparable AUC (0.735) with improved sensitivity. Across regression models, cross‐validated R^2^ values were near‐zero or negative, indicating limited ability to predict the exact magnitude of overbite change from baseline variables alone. SHAP ranked pre‐treatment overbite, ramus height, upper occlusal plane to SN angle, sella‐to‐condylion distance, and condylar positional indices among the most influential predictors for classification.

**Conclusion:**

Interpretable ML enables improved risk stratification for clinically meaningful anterior overbite decrease after stabilization splint therapy in TMD patients, whereas predicting the exact magnitude of overbite change remains limited using pre‐treatment cephalometrics alone. These models may support individualized counseling and closer monitoring for patients at higher risk of open‐bite development during splint therapy.

## 1 Introduction

Temporomandibular disorder (TMD) is a common condition characterized by pain and dysfunction of the masticatory system, affecting approximately 5%–12% of the general population (Schiffman et al. 2014; Scrivani et al. 2008). Stabilization splint treatment (ST) is widely used as a conservative management approach for TMDs, aiming to establish an orthopedically stable joint position and reduce abnormal muscle activity (Okeson 2019). Despite its well‐established efficacy for pain reduction and joint stabilization (Widmalm 1999; Fricton 2006), a well‐documented side effect of ST is the development of anterior open‐bite, which occurs when the anterior overbite decreases during or after treatment (Todd and Freer 1994; Nissani 2001; Magdaleno and Ginestal 2010).

Oh et al. (2022) previously investigated dentoskeletal factors that could predict anterior open‐bite development after ST using conventional statistical methods, including Kruskal–Wallis tests, Spearman correlation analysis, receiver operating characteristic (ROC) curve analysis, and multiple regression analysis. Their study identified facial height ratio (FHR), ramus height, and sella to condylion distance as significant predictors. However, the multiple regression model achieved an R‐squared value of 0.135, indicating that the identified factors explained 13.5% of the variance in overbite changes. The ROC analysis yielded sensitivity and specificity values ranging from 0.55 to 0.73, highlighting the potential for further improvement in individual patient prediction.

In recent years, machine learning (ML)—computational methods that learn predictive patterns from data without requiring all relationships to be specified a priori—has demonstrated substantial potential in medical and dental prediction tasks (Rajkomar et al. 2019; Hung et al. 2019; Lee et al. 2018). Unlike conventional statistical methods that often model prespecified relationships, ML algorithms can capture complex nonlinear patterns and interactions among multiple features (Chen and Guestrin 2016). Several studies have successfully applied ML to TMD diagnosis and classification (Cui et al. 2024; Yıldız et al. 2024; Wu et al. 2025), orthodontic treatment planning (Albalawi and Abalkhail 2022; Alqerban 2024), and malocclusion prediction (Li et al. 2019; Taraji et al. 2023). Ensemble methods, which combine multiple decision trees or sequential learners to improve prediction, include random forest and gradient boosting algorithms such as XGBoost and LightGBM, and have shown favorable performance in clinical prediction tasks (Breiman 2001; Ke et al. 2017).

Furthermore, explainable artificial intelligence (XAI) methods, particularly SHapley Additive exPlanations (SHAP) (Lundberg and Lee 2017), can make otherwise complex ML models more interpretable. SHAP assigns each feature a value representing how much that feature shifts a model prediction relative to a reference prediction. Thus, SHAP can provide both global feature‐importance rankings and patient‐level explanations, helping clinicians understand which variables contribute to model predictions.

However, to the best of our knowledge, no study has applied ML algorithms to predict anterior open‐bite development after ST in TMD patients. Moreover, no study has systematically compared the predictive performance of ML methods with conventional statistical approaches using the same clinical dataset. Therefore, the aims of this study were: (1) to evaluate whether ML algorithms can achieve superior predictive performance compared with conventional statistical methods for classifying patients at risk of anterior open‐bite development after ST; (2) to assess the feasibility of predicting the magnitude of overbite changes using ML regression models; and (3) to identify and interpret the most important dentoskeletal predictors using SHAP analysis, comparing these with the findings from our prior conventional analysis.

## 2 Materials and Methods

### 2.1 Subjects and Data

This study utilized the same dataset from the previously published study by Oh et al. (2022). Eighty‐seven TMD patients (mean age: 39.1 ± 15.6 years; 68 females and 19 males) who attended the Department of Oral Medicine, Pusan National University Dental Hospital, between January 2015 and December 2019 were included. Inclusion criteria were: (1) diagnosis of TMD according to the Diagnostic Criteria for Temporomandibular Disorders (DC/TMD); (2) stable occlusal conditions, defined as at least one pair of central incisors, one pair of premolars, and one pair of molars occluding with opposing teeth on both the left and right sides; and (3) age > 20 years. Exclusion criteria were: (1) previous orthodontic treatment and (2) systemic diseases affecting the skeletal system. All patients were treated with ST, physical therapy, and pharmacological therapy. The study was approved by the institutional review board of Pusan National University Dental Hospital (IRB No. PNUDH‐2019‐030).

### 2.2 Cephalometric Measurements

Lateral cephalometric images were obtained from computed tomography (CT) scans before and after ST. A total of 35 pre‐treatment features were extracted, comprising 12 dental measurements (U1 to SN, L1 to MP, R U1VD, R L1VD, R U6VD, R L6VD, interincisal angle, Spee curve depth, U1 to A‐Pog, L1 to A‐Pog, overjet, and overbite), 22 skeletal measurements (APDI, ODI, Bjork sum, FMA, FHR, Y‐axis, mandibular plane angle, facial angle, base plane angle, lower anterior facial height, upper and lower occlusal plane to SN, SNA, SNB, ANB, Wits appraisal, ramus height, mandibular body length, angle of convexity, sella to condylion, condylion to SN plane, and condylion‐SN perpendicular distance), and patient age. All cephalometric landmarks and measurements were as defined in Tables 1 and 2 of our prior study (Oh et al. 2022).

### 2.3 Outcome Variables

The primary outcome was the change in anterior overbite after ST, calculated as the overbite after ST minus the overbite before ST (overbite difference, OD). For the classification task, patients were categorized into two groups based on the OD: (1) Decrease group (OD < −0.5 mm; *n* = 37), representing patients who developed a clinically meaningful decrease in anterior overbite (open‐bite tendency); and (2) Non‐Decrease group (OD > = −0.5 mm; *n* = 50), combining patients with no change and those with increased overbite. This binary classification was chosen because the clinical priority is identifying patients at risk of open‐bite development, and the original three‐group classification (Decrease/No Change/Increase) resulted in a severely imbalanced Increase group (*n* = 8). For the regression task, the continuous OD value was used as the target variable.

### 2.4 Data Preprocessing

Missing values were present in three features (SNB, condylion to SN plane, and condylion‐SN perpendicular distance; 1.1% each) and were imputed using median values. All features were standardized to a zero mean and unit variance using z‐score normalization prior to model training. No features were excluded based on missing data thresholds. Outliers were identified using the interquartile range (IQR) method, but were retained in the dataset to preserve the clinical representativeness of the sample. In this context, standardization places variables measured on different scales onto a comparable scale, while imputation replaces the small number of missing observations using information from the available data.

### 2.5 ML Classification Models

Six classification algorithms were employed to predict the binary outcome (Decrease vs. Non‐Decrease):

1. Logistic regression (LR), serving as the baseline model analogous to the conventional statistical approach, with class weight balancing;
2. Random forest (RF), an ensemble of decision trees with 100 estimators and balanced class weights;
3. Extreme gradient boosting (XGBoost), with 100 estimators and scale_pos_weight adjustment for class imbalance;
4. Light gradient boosting machine (LightGBM), with 100 estimators and is_unbalance parameter;
5. Support vector machine (SVM) with radial basis function (RBF) kernel and balanced class weights;
6. k‐nearest neighbors (k‐NN) with *k* = 5.

To address the class imbalance (37 Decrease vs. 50 Non‐Decrease), three strategies were employed: (1) class‐weight adjustment within the algorithms; (2) Synthetic Minority Over‐sampling Technique (SMOTE) (Chawla 2002; Batista 2004), which generates synthetic examples of the minority class, with k_neighbors = 3 and applied only within the training portion of each cross‐validation loop to prevent data leakage; and (3) binary reclassification of the original three groups.

### 2.6 ML Regression Models

Nine regression algorithms were employed to predict the continuous OD value: linear regression, Lasso regression (alpha = 0.1), Ridge regression (alpha = 1.0), ElasticNet (alpha = 0.1, l1_ratio = 0.5), random forest regressor (100 estimators), gradient boosting regressor (100 estimators), XGBoost regressor (100 estimators), LightGBM regressor (100 estimators), and support vector regression (SVR) with RBF kernel.

### 2.7 Model Validation

Two cross‐validation strategies were employed to estimate how well the models would perform on unseen patients. Cross‐validation repeatedly separates the available data into training and test subsets, thereby providing an internal estimate of out‐of‐sample performance:

1. Stratified fivefold cross‐validation (5‐fold CV): The dataset was divided into five folds with preserved class proportions. Each fold served as the test set once, while the remaining four folds were used for training. This process was repeated five times, and performance metrics were computed on the aggregated predictions.
2. Leave‐one‐out cross‐validation (LOOCV): Each sample served as the test set once, while the remaining 86 samples were used for training. This approach provides the least biased performance estimate and is particularly suitable for small datasets (Lundberg and Lee 2017).

### 2.8 Performance Metrics

Classification performance was assessed using accuracy, precision, recall (sensitivity), F1‐score, specificity, and area under the receiver operating characteristic curve (AUC‐ROC). Regression performance was assessed using the coefficient of determination (R‐squared), root mean squared error (RMSE), and mean absolute error (MAE). These metrics were compared directly with the results reported in our prior study (Oh et al. 2022).

### 2.9 Feature Importance and Model Interpretation

**Figure 1:**
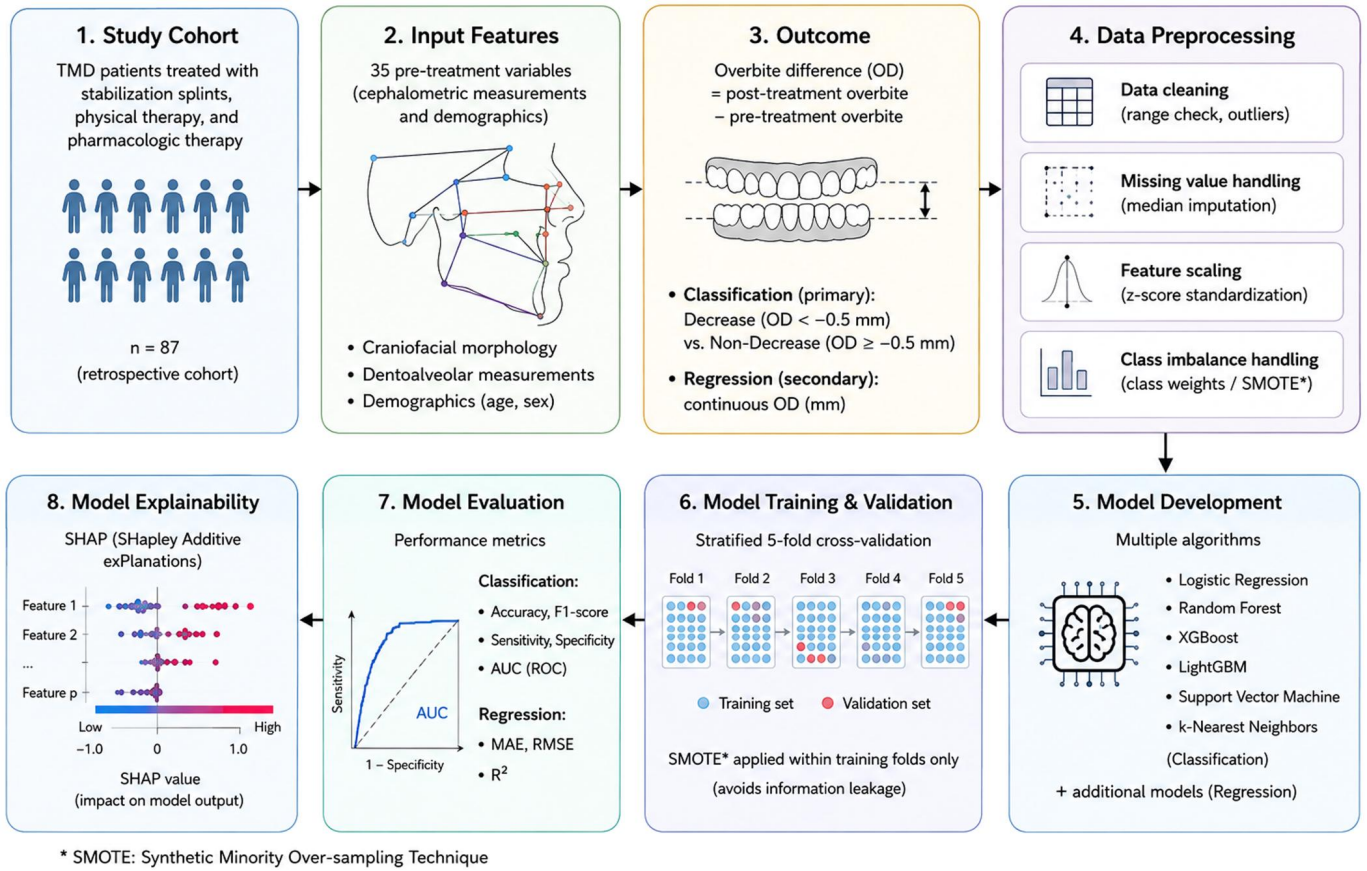
Machine learning workflow for prediction of anterior open‐bite development after stabilization splint treatment in patients with temporomandibular disorder. The workflow summarizes cohort selection, pre‐treatment input features, outcome definition, data preprocessing, model development, internal validation, performance evaluation, and SHAP‐based model explainability. SMOTE, Synthetic Minority Over‐sampling Technique.

Three complementary approaches were used to assess feature importance:

1. Random forest feature importance, based on the mean decrease in Gini impurity across all trees;
2. XGBoost feature importance (gain), measuring the average improvement in the loss function contributed by each feature;
3. SHAP analysis (Breiman 2001), which decomposes individual predictions into additive contributions from each feature based on cooperative game theory. SHAP values were computed using TreeExplainer for the XGBoost classifier and regressor. The mean absolute SHAP value for each feature was used as the global importance metric. SHAP summary plots were generated to visualize both feature importance and the direction of feature effects. The overall ML workflow, including data preprocessing, model development, validation, performance evaluation, and SHAP‐based interpretation, is summarized in Figure 1.

### 2.10 Statistical Software

All ML analyses were performed using Python 3.14.0 with scikit‐learn (version 1.8.0), XGBoost (version 3.2.0), LightGBM (version 4.6.0), imbalanced‐learn (version 0.14.1), and SHAP (version 0.50.0). Visualizations were generated using matplotlib (version 3.10.8) and seaborn (version 0.13.2).

## 3 Results

### 3.1 Descriptive Statistics

**Figure 2:**
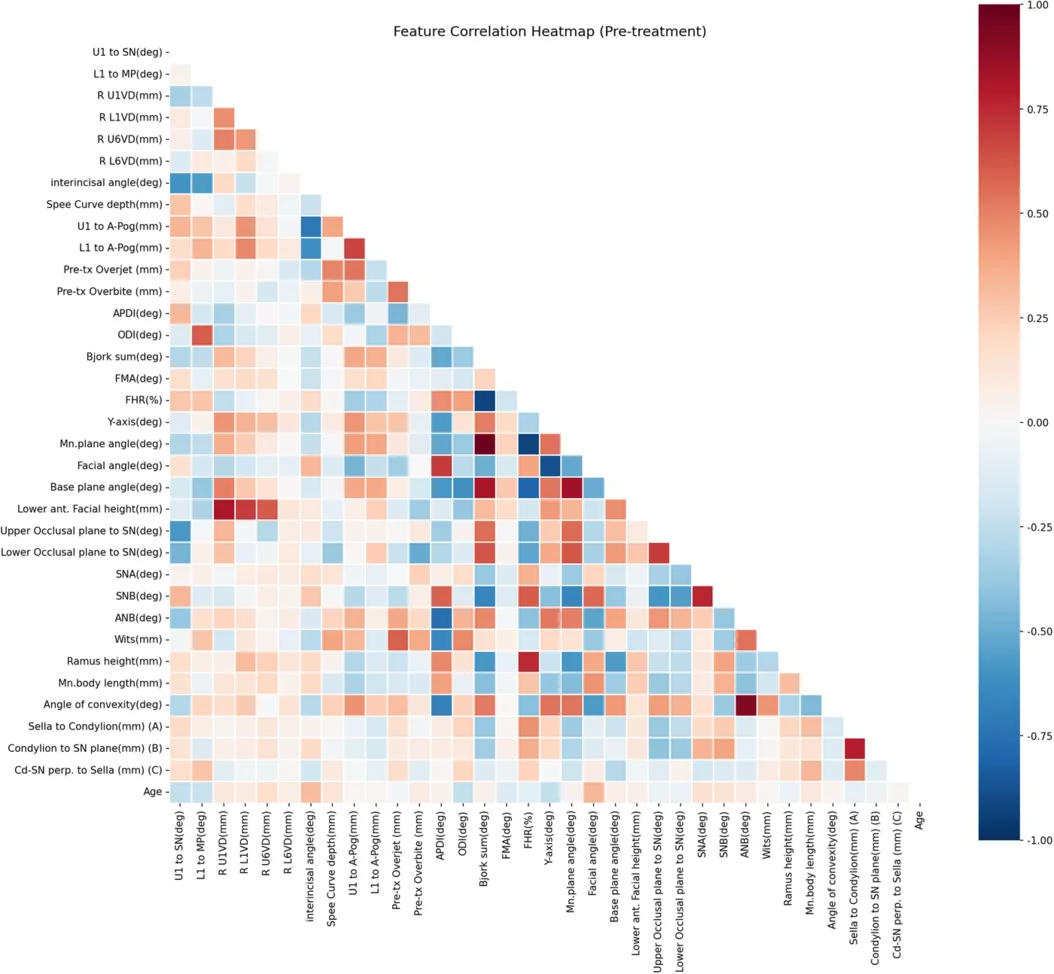
Feature correlation heatmap of pre‐treatment dentoskeletal measurements. The lower triangle of the Pearson correlation matrix is displayed with a diverging color scheme (red = positive, blue = negative).

The group‐wise descriptive statistics for key pre‐treatment dentoskeletal variables are presented in Table 1. Compared with the No Change group, the Decrease group exhibited lower FHR (59.54% vs. 62.17%), shorter ramus height (40.57 vs. 44.43 mm), higher U1 to A‐Pog (9.47 vs. 7.53 mm), and higher ANB (4.92° vs. 3.66°), supporting the presence of a recognizable pre‐treatment morphologic profile associated with clinically meaningful anterior overbite decrease. The correlation structure among the pre‐treatment dentoskeletal features is illustrated in Figure 2.

**Table 1:** Group‐wise descriptive statistics for key dentoskeletal variables before stabilization splint treatment (mean ± SD).

Variable	Decrease (*n* = 37)	No Change (*n* = 42)	Increase (*n* = 8)
FHR (%)	59.54 ± 5.45	62.17 ± 4.84	63.25 ± 4.94
Ramus height (mm)	40.57 ± 5.20	44.43 ± 5.37	44.31 ± 5.33
U1 to A‐Pog (mm)	9.47 ± 3.20	7.53 ± 2.36	8.65 ± 2.46
Interincisal angle (deg)	122.55 ± 11.48	127.40 ± 9.40	121.58 ± 9.34
ANB (deg)	4.92 ± 1.98	3.66 ± 2.21	3.84 ± 2.34
Wits (mm)	0.77 ± 3.50	−1.68 ± 3.38	0.04 ± 1.88
Sella to Condylion (mm)	20.71 ± 2.89	22.10 ± 4.07	24.09 ± 3.54
Pre‐Tx Overbite (mm)	2.88 ± 2.36	1.76 ± 1.17	1.90 ± 2.65

### 3.2 Classification Performance

Table 2 summarizes binary classification performance (Decrease vs. Non‐Decrease) using stratified fivefold cross‐validation. XGBoost achieved the best overall discrimination (AUC = 0.734) with accuracy 0.701 and F1‐score 0.658, outperforming logistic regression (AUC = 0.611; accuracy 0.552; F1‐score 0.506). Other tree‐based ensemble classifiers performed similarly well (LightGBM AUC = 0.721; random forest AUC = 0.717), and SVM also exceeded logistic regression (AUC = 0.697), whereas k‐NN demonstrated lower discrimination (AUC = 0.652). Collectively, these results indicate that ensemble models leveraging nonlinear interactions among cephalometric variables yield improved risk stratification compared with a linear baseline in this dataset.

**Table 2:** Binary classification performance (Decrease vs. Non‐decrease) with stratified fivefold cross‐validation.

Model	Accuracy	Precision	Recall	F1‐score	AUC
Logistic regression	0.552	0.476	0.541	0.506	0.611
Random forest	0.655	0.630	0.459	0.531	0.717
XGBoost	0.701	0.641	0.676	0.658	0.734
LightGBM	0.632	0.556	0.676	0.610	0.721
SVM (RBF)	0.690	0.632	0.649	0.640	0.697
k‐NN	0.644	0.615	0.432	0.508	0.652

LOOCV (Supplementary Table S1) showed consistent patterns but generally lower performance than fivefold CV, reflecting higher variance with leave‐one‐out estimation in small samples. Under LOOCV, XGBoost maintained the highest F1‐score (0.521) with sensitivity 0.514 and specificity 0.660, whereas random forest achieved the highest specificity (0.780) and AUC (0.686) but with lower sensitivity (0.351), highlighting a clinically relevant trade‐off between avoiding false positives versus minimizing false negatives.

When class imbalance was addressed using SMOTE (Supplementary Table S2), sensitivity improved for ensemble methods. Random forest + SMOTE achieved the highest AUC (0.735) (accuracy = 0.632, F1 = 0.556, sensitivity = 0.541, specificity = 0.700), while XGBoost + SMOTE achieved the highest sensitivity (0.622) and F1‐score (0.582) with AUC 0.705, consistent with a screening‐oriented profile that prioritizes detection of at‐risk patients.

### 3.3 Three‐Class Classification

Three‐class classification (Decrease/No Change/Increase) showed markedly reduced performance across models (Supplementary Table S3), consistent with severe class imbalance (only 8 patients in the Increase group). Random forest achieved the best overall results (accuracy 0.598; weighted F1 0.567), supporting the rationale for focusing on the binary Decrease vs Non‐Decrease framework as the more stable and clinically interpretable prediction task.

### 3.4 Regression Performance

Table 3 presents regression results for predicting continuous OD magnitude. Under fivefold cross‐validation, all models yielded negative R^2^ values; Lasso regression performed closest to zero (R^2^ = −0.026, RMSE = 1.073, MAE = 0.786). Under LOOCV, only Lasso regression achieved a marginally positive R^2^ (0.025) while all other models remained negative. Compared with the in‐sample exploratory regression from our prior study (R^2^ = 0.135) (Oh et al. 2022), these cross‐validated results provide a more conservative estimate of generalizable performance and indicate that precise magnitude prediction is limited using pre‐treatment cephalometrics alone.

**Table 3:** Regression performance for predicting overbite difference (mm).

Model	R‐squared (5‐CV)	RMSE (5‐CV)	R‐squared (LOOCV)	RMSE (LOOCV)
Linear regression	−13.378	4.017	—	—
Lasso (alpha = 0.1)	−0.026	1.073	0.025	1.046
Ridge (alpha = 1.0)	−0.519	1.306	−0.504	1.299
ElasticNet	−0.042	1.081	—	—
Random forest	−0.048	1.085	−0.088	1.105
Gradient boosting	−0.140	1.131	—	—
XGBoost	−0.410	1.258	−0.449	1.275
LightGBM	−0.188	1.154	−0.193	1.157
SVR	−0.065^a^	1.093	—	—
Prior study (Oh et al. 2022)	0.135^a^	N/A	—	—

^a^ In‐sample R‐squared (exploratory regression).

### 3.5 Feature Importance Analysis

**Figure 3:**
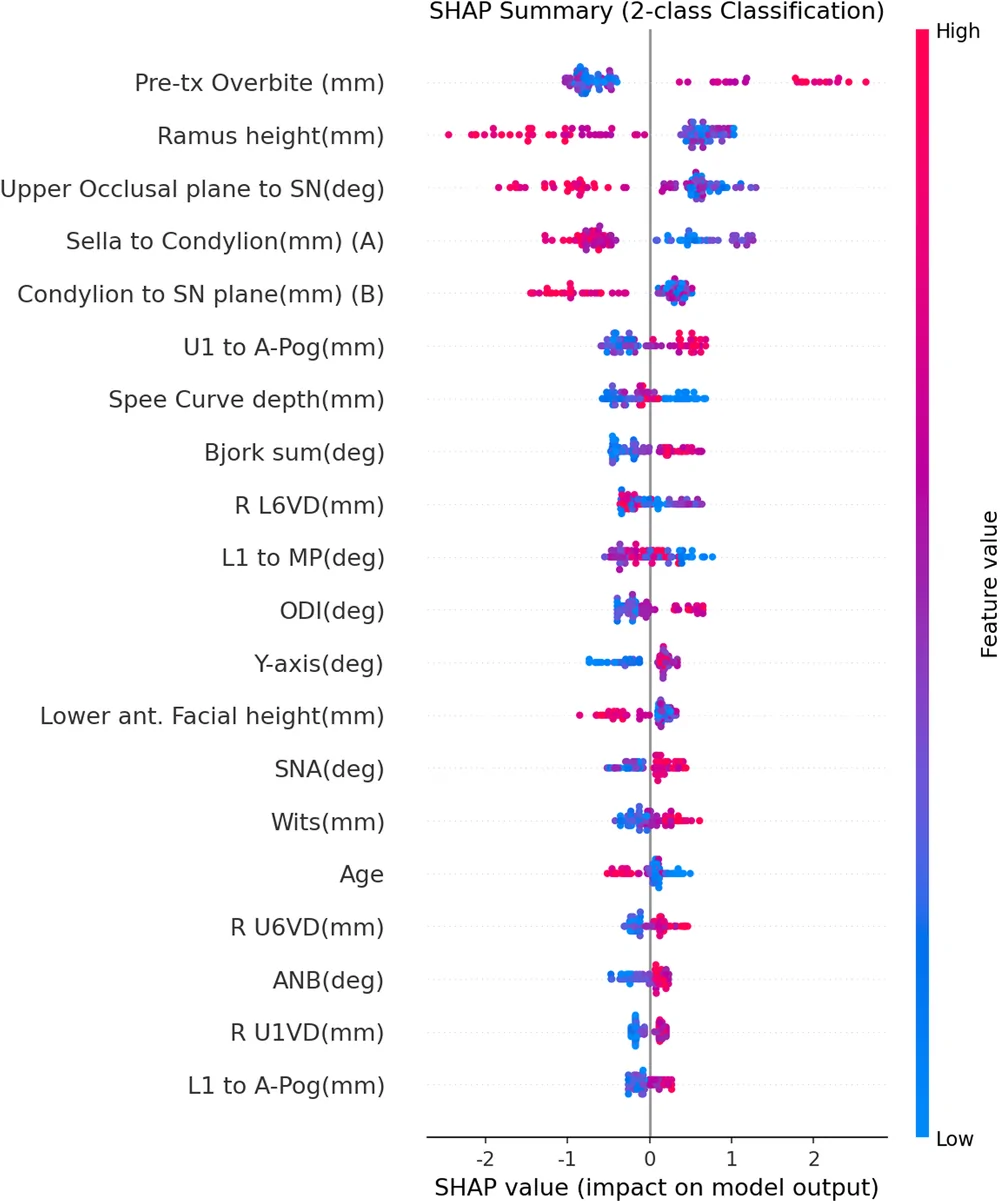
SHAP summary plot for XGBoost binary classification. Each dot represents a patient. The *x*‐axis shows the SHAP value (feature contribution to the model output for the predicted class), and color denotes the feature value (red = higher value, blue = lower value).

Table 4 summarizes SHAP‐based feature importance for classification and regression tasks and compares them with significant predictors from our prior study. For classification, the five most influential features (Figure 3 and Supplementary Figure S1) were pre‐treatment overbite (mean |SHAP | 0.961), ramus height (0.890), upper occlusal plane to SN (0.768), sella‐to‐condylion distance (0.700), and condylion‐to‐SN plane distance (0.518). For regression (Supplementary Figure S2), the most influential predictors were condylion‐to‐SN plane distance (0.203), pre‐treatment overbite (0.120), sella‐to‐condylion distance (0.114), lower anterior facial height (0.088), and ramus height (0.075), suggesting that predictors contributing to binary threshold crossing differ from those contributing to the continuous OD estimate.

**Table 4:** Feature importance comparison: SHAP values versus prior study predictors.

Rank	SHAP (Classification)	Mean | SHAP |	SHAP (Regression)	Mean | SHAP |	Prior study (Oh et al. 2022)
1	Pre‐Tx overbite	0.961	Condylion to SN plane	0.203	Ramus height^a^
2	Ramus height	0.890	Pre‐Tx Overbite	0.120	Condylion to SN plane
3	Upper Occ. plane to SN	0.768	Sella to Condylion	0.114	U1 to A‐Pog^a^
4	Sella to Condylion	0.700	Lower ant. facial height	0.088	Facial angle
5	Condylion to SN plane	0.518	Ramus height	0.075	Interincisal angle
6	U1 to A‐Pog	0.371	Y‐axis	0.074	—
7	Spee Curve depth	0.306	R U6VD	0.067	—
8	Bjork sum	0.303	Pre‐Tx Overjet	0.063	—
9	R L6VD	0.273	Condylion‐SN perp.	0.061	—
10	L1 to MP	0.268	Bjork sum	0.059	—

^a^ Statistically significant in prior study.

Of the seven predictors previously identified as significant (U1 to A–Pog, FHR, ANB, Wits, ramus height, angle of convexity, and sella‐to‐condylion), four were confirmed within the ML classification top 10 (ramus height, sella‐to‐condylion, condylion‐to‐SN plane, U1 to A–Pog). Notably, ML identified pre‐treatment overbite as the most influential classification predictor (mean |SHAP | 0.961), consistent with its observed baseline group difference in our prior study (*p* = 0.017) but not included in the prior regression model. Additionally, the upper occlusal plane to SN, Bjork sum, and Spee curve depth emerged as newly prominent predictors not highlighted in our previous conventional analysis.

## 4 Discussion

This study is the first to apply ML algorithms for predicting anterior open‐bite development after ST in TMD patients, and to systematically compare ML performance with conventional statistical methods using the same clinical dataset. The principal findings were: (1) XGBoost classification achieved an AUC of 0.734, representing a substantial improvement over the conventional ROC analysis; (2) regression prediction of the continuous overbite change was not feasible with the current feature set, regardless of the modeling approach; and (3) SHAP analysis both confirmed and extended our prior study's findings regarding important dentoskeletal predictors.

The superior performance of XGBoost and other ensemble methods over logistic regression in this study is consistent with findings from recent ML applications in TMD research. Cui et al. (2024) reported that random forest achieved an AUC of 0.857 for TMD prediction in a study of 949 adults, and Yildiz et al. (2024) found that bagging algorithms achieved an AUC of 0.939 for TMD classification using clinical parameters. While our AUC values were lower, this reflects the inherently more challenging prediction task (treatment outcome rather than disease presence) and the smaller sample size (*n* = 87). The improvement from AUC ~ 0.65–0.734 is nevertheless clinically meaningful, as it approaches the threshold of 0.75 considered acceptable for clinical screening tools (Mandrekar 2010).

In the broader context of ML applications in orthodontics and occlusal prediction, Alqerban (2024) recently applied ML for treatment decisions in anterior open‐bite orthodontic cases, demonstrating the feasibility of data‐driven approaches in open‐bite management. Similarly, Bianchi et al. (2020) showed that ML combined with biomarkers could diagnose TMJ osteoarthritis earlier than conventional methods. Wu et al. (2025) provided a comprehensive overview of ML applications in TMD diagnosis, highlighting that ensemble methods consistently outperform single classifiers. Our findings align with this growing body of evidence supporting the utility of ML in craniomandibular research.

It is worth noting that Christodoulou et al. (2019) conducted a systematic review comparing ML models with logistic regression across 71 clinical prediction studies, concluding that ML did not consistently outperform logistic regression, especially with smaller datasets. In our study, logistic regression yielded the lowest AUC (0.611), while ensemble methods (XGBoost, RF, and LightGBM) achieved AUCs of 0.717–0.734, suggesting that the nonlinear interactions among dentoskeletal variables provide meaningful predictive information that linear models cannot capture. This distinction may be attributable to the complex biomechanical relationships between craniofacial structures, where multiple skeletal and dental factors interact to influence treatment outcomes (El‐Dawlatly et al. 2015).

Stabilization splints are widely used in TMD management; however, a subset of patients experiences clinically relevant anterior overbite decrease (open‐bite tendency), which may require earlier recognition and closer monitoring. Our results suggest that interpretable ensemble ML models can support risk‐based follow‐up planning, where patients predicted as high risk could undergo shorter recall intervals, more frequent occlusal checks, and earlier consideration of alternative management strategies, while low‐risk patients may follow standard follow‐up schedules.

An important finding of this study was that all ML regression models yielded near‐zero or negative R‐squared values under cross‐validation. Our prior study (Oh et al. 2022) reported an in‐sample R‐squared of 0.135, which demonstrated meaningful associative relationships between dentoskeletal factors and overbite changes. The present study extends this work by evaluating out‐of‐sample predictive accuracy through cross‐validation, as recommended by the TRIPOD guidelines (Collins et al. 2015). The cross‐validated results indicate that while the identified dentoskeletal factors are meaningfully associated with overbite change direction, predicting the exact magnitude from pre‐treatment cephalometric variables alone is inherently limited. This likely reflects the influence of dynamic factors during treatment, such as condylar remodeling processes (Ok et al. 2016, 2014) and individual biological variability, which are not captured by pre‐treatment measurements. These findings suggest that the binary classification approach (identifying at‐risk patients) is more clinically achievable and pragmatic than attempting precise quantitative predictions.

The SHAP analysis (Lundberg and Lee 2017) provided clinically interpretable feature effects and indicated that risk stratification reflects a combination of dentoalveolar and vertical/condylar morphology rather than a single determinant. Pre‐treatment overbite was the strongest classification feature (mean |SHAP | = 0.961), but this should not be interpreted as evidence of a causal mechanism. Because the classification outcome is defined by crossing a fixed change threshold, baseline overbite can have substantial predictive utility for group assignment. In contrast, its lower importance in continuous regression (mean |SHAP | = 0.120) suggests that baseline overbite alone does not determine the magnitude of treatment‐related change. Accordingly, the classification result is best interpreted as a threshold‐related predictive signal acting within a multivariable morphologic profile.

Second, the ML models confirmed ramus height and sella to condylion distance as important predictors, consistent with our prior study's finding that short posterior facial height and condyle position near the cranial base are predisposing factors for open‐bite development after ST. These findings are also consistent with the known skeletal characteristics of anterior open‐bite, including a steep mandibular plane angle, increased gonial angle, and short posterior facial height (El‐Dawlatly et al. 2015; Miyawaki et al. 2005; Wajid et al. 2018; Cangialosi 1984). Miyawaki et al. (2005) further demonstrated that occlusal force and condylar motion patterns differ significantly in anterior open‐bite patients, supporting the role of these skeletal factors. The SHAP dependency analysis revealed that these relationships are approximately linear, supporting our prior study's interpretation. However, the ML models additionally identified Bjork sum, upper occlusal plane to SN angle, and Spee curve depth as important features. These variables characterize the overall craniofacial growth pattern and dental arch morphology, suggesting that open‐bite development after ST may be influenced by a broader set of dentoskeletal factors than previously recognized.

Third, the condylion to SN plane distance emerged as the most important predictor in the regression task (mean |SHAP | = 0.203), consistent with its role in the prior regression equation. This variable reflects the vertical position of the condyle relative to the cranial base, and its importance supports the hypothesis that condylar position changes during ST may be a key mechanism underlying open‐bite development (Ok et al. 2016, 2014). Ok et al. (2016) previously reported glenoid fossa remodeling during ST, and Ok et al. (2014) observed anterior condylar remodeling in TMJ osteoarthritis patients treated with stabilization splints, both of which may contribute to changes in the vertical dimension.

The application of SMOTE (Chawla et al. 2002) improved classification sensitivity (from 0.459 to 0.541 for random forest) at a modest cost to specificity (from 0.717 to 0.700 in AUC‐equivalent terms). Chawla et al. (2002) originally demonstrated that SMOTE effectively reduces bias toward the majority class, and Krawczyk (2016) further discussed the challenges of class imbalance in medical domains with small samples, recommending the combination of SMOTE with ensemble methods. Our results confirm this recommendation, as the random forest with SMOTE achieved the highest overall AUC (0.735). Given that the clinical consequence of missing an at‐risk patient (false negative) is more serious than an unnecessary precaution for a non‐at‐risk patient (false positive), the SMOTE‐enhanced model may be preferable in clinical practice.

Several limitations should be acknowledged. First, the sample size of 87 patients is relatively small for ML model development, particularly in relation to the 35 candidate predictors. This increases the risk of overfitting and may make feature rankings, decision boundaries, and performance estimates sensitive to the specific composition of the sample. Although fivefold cross‐validation and LOOCV were used to obtain internal estimates of out‐of‐sample performance, cross‐validation cannot substitute for validation in an independent cohort. The observed AUCs should therefore be regarded as preliminary estimates, and the apparent differences between algorithms may not remain stable in larger samples. The small Increase subgroup (*n* = 8) also limited reliable three‐class modeling and motivated the clinically oriented binary outcome. Second, this was a retrospective analysis of an existing single‐center dataset; prospective external validation in larger, preferably multi‐center cohorts is needed before clinical implementation and to assess transportability to populations with different demographic, diagnostic, or treatment characteristics. Third, the feature set was limited primarily to baseline cephalometric measurements; inclusion of additional clinical variables (e.g., parafunctional habits, muscle characteristics, treatment adherence, psychosocial factors, and treatment‐related changes) may improve predictive performance. Fourth, three‐dimensional cephalometric measurements or condylar morphology features from cone‐beam CT could provide additional predictive information not captured by the current measurements. Finally, SHAP values describe how features contribute to predictions within the fitted model and should not be interpreted as causal effects.

Future studies should focus on: (1) external validation with larger, multi‐center datasets as recommended by the TRIPOD guidelines (Collins et al. 2015); (2) incorporation of additional clinical and imaging features, including three‐dimensional morphological data; (3) development of a user‐friendly clinical decision support tool based on the ML classification model, as advocated by recent reviews on AI in orthodontics (Albalawi and Abalkhail 2022); and (4) longitudinal studies to validate the identified risk factors prospectively and to monitor the long‐term predictive value of the ML models.

## 5 Conclusions

1. ML classification, particularly XGBoost (AUC = 0.734), demonstrated superior performance compared with conventional ROC analysis (AUC ~ 0.65) for predicting anterior open‐bite development after ST in TMD patients.
2. Binary classification (Decrease vs. Non‐Decrease) is a more clinically feasible and achievable predictive task than continuous regression of overbite change magnitude. Cross‐validation analysis confirmed that predicting the exact magnitude of overbite change from pre‐treatment variables alone is inherently limited, supporting the clinical utility of binary risk classification.
3. SHAP analysis confirmed ramus height, sella to condylion, and U1 to A‐Pog as important predictors, consistent with our prior conventional analysis. Additionally, pre‐treatment overbite was identified as the strongest single predictor, and upper occlusal plane to SN, Bjork sum, and Spee curve depth were identified as newly important variables.
4. The combination of ensemble ML algorithms with SHAP‐based interpretation provides both improved predictive performance and clinically interpretable insights, supporting its potential integration into clinical decision‐making for TMD management.
5. Prospective validation with larger datasets is needed to confirm the generalizability of these findings and to develop a clinical decision support tool.

## Author Contributions

**Jae Joon Hwang** conceptualization, methodology, investigation, data curation, writing – original draft, writing – review and editing. **Hye‐Mi Jeon:** methodology, investigation, data curation, writing – review and editing. **Hye‐Min Ju:** formal analysis, visualization, writing – review and editing. **Sung‐Hee Jeong:** investigation, methodology, writing – review and editing. **Yong‐Woo Ahn:** conceptualization, supervision, writing – review and editing. **Soo‐Min Ok:** conceptualization, methodology, investigation, formal analysis, data curation, writing – original draft, writing – review and editing, visualization, supervision, project administration.

## Ethics Statement

This study was approved by the institutional review board of Pusan National University Dental Hospital (IRB No. PNUDH‐2019‐030).

## Consent

Informed consent was waived by the IRB due to the retrospective design.

## Conflicts of Interest

The authors declare no conflicts of interest.

## Supporting information


Supporting File 1

Supporting File 2

Supporting File 3

## Data Availability

The data that support the findings of this study are available from the corresponding author upon reasonable request. Restrictions apply to the availability of these data, which were used under institutional review board approval for this study.
