# Supplementary material for: Machine Learning Prediction of Anterior Open‐Bite Development After Stabilization Splint Treatment in Temporomandibular Disorder
Source: Clin Exp Dent Res. 2026 Aug 30;12(5):e70447. doi: 10.1002/cre2.70447 (PMC13527398; doi:10.1002/cre2.70447)
Supplement: Supplementary file 1 [file CRE2-12-e70447-s002.docx]

Supplementary Table S1. Binary classification performance (Decrease vs Non-Decrease) under LOOCV. Reported metrics include accuracy, F1-score, sensitivity, specificity, and AUC for each classifier.

| **Model** | **Accuracy** | **F1-score** | **Sensitivity** | **Specificity** | **AUC** |
| --- | --- | --- | --- | --- | --- |
| Logistic Regression | 0.586 | 0.500 | 0.486 | 0.660 | 0.643 |
| Random Forest | 0.598 | 0.426 | 0.351 | 0.780 | 0.686 |
| XGBoost | 0.598 | 0.521 | 0.514 | 0.660 | 0.674 |

Supplementary Table S2. Binary classification performance under stratified 5-fold cross-validation with SMOTE applied within training folds. Metrics include accuracy, F1-score, sensitivity, specificity, and AUC.

| **Model** | **Accuracy** | **F1-score** | **Sensitivity** | **Specificity** | **AUC** |
| --- | --- | --- | --- | --- | --- |
| Logistic Regression + SMOTE | 0.609 | 0.528 | 0.514 | 0.680 | 0.609 |
| Random Forest + SMOTE | 0.632 | 0.556 | 0.541 | 0.700 | 0.735 |
| XGBoost + SMOTE | 0.621 | 0.582 | 0.622 | 0.620 | 0.705 |

Supplementary Table S3. Three-class classification performance (Decrease/No Change/Increase) under stratified 5-fold cross-validation. Metrics include accuracy, macro F1, and weighted F1.

| **Model** | **Accuracy** | **F1 (macro)** | **F1 (weighted)** |
| --- | --- | --- | --- |
| Logistic Regression | 0.448 | 0.380 | 0.463 |
| Random Forest | 0.598 | 0.415 | 0.567 |
| LightGBM | 0.563 | 0.395 | 0.541 |

Supplementary Figure S1

**Supplementary Figure S1.** Global SHAP feature importance for binary classification (Decrease vs Non-Decrease).

Bar plot of mean absolute SHAP values (mean |SHAP|) for the XGBoost classifier, representing the global magnitude of each feature’s contribution to the binary prediction regardless of direction. Higher mean |SHAP| indicates a larger average impact on the model output across patients. The ranking highlights that binary risk stratification for clinically meaningful anterior overbite decrease after stabilization splint therapy is driven by a combination of (i) baseline dentoalveolar status (e.g., pre-treatment overbite) and (ii) vertical/condylar positional and posterior facial height–related measures (e.g., ramus height, sella-to-condylion distance, condylion-to-SN plane distance), supporting a multivariable morphologic profile rather than a single determinant.

Supplementary Figure S2

**Supplementary Figure S2.** SHAP summary plot for XGBoost regression predicting continuous overbite difference (OD, mm).

SHAP summary plot for the XGBoost regression model predicting continuous OD (post–pre overbite, mm). Each dot represents one patient. The x-axis denotes SHAP values (feature contributions to the predicted continuous OD), where positive SHAP values shift predictions toward larger OD and negative values shift toward smaller OD. Dot color reflects the feature value (red = higher value; blue = lower value), enabling visualization of directionality (i.e., whether higher or lower feature values tend to increase or decrease the predicted OD). In contrast to the classification task where pre-treatment overbite dominated global importance, the regression task showed greater influence of condylar positional indices (e.g., condylion-to-SN plane distance), consistent with the observed limitation of predicting exact OD magnitude from baseline cephalometrics alone under cross-validation.
